# The *GPX3* rs3828599 genotype in combination with alcohol consumption and age helps identify a high-risk hypertension subgroup for antioxidant intervention

**DOI:** 10.1186/s12967-025-07243-2

**Published:** 2025-10-28

**Authors:** Zhongjie Xie, Baogang Wu, Yanli Chen, Yingxian Sun, Ying Hao

**Affiliations:** 1 Department of Geriatrics, Taizhou First People’s Hospital, The First Affiliated People’s Hospital of Taizhou University, Taizhou, Zhejiang 318020 China; 2 Department of Geriatrics, Taizhou First People’s Hospital, The First Affiliated People’s Hospital of Taizhou University, 218 Hengjie Road, Huangyan District, Taizhou, Zhejiang 318020 China; 3 Department of Cardiology, Sheng Jing Hospital of China Medical University, Shenyang, China; 4 Department of Cardiology, The First Hospital of China Medical University, Shenyang, China

**Keywords:** Hypertension, *GPX3* polymorphism, Gene environment interaction, Precision medicine, Oxidative stress, Antioxidant therapy

## Abstract

**Background:**

Essential hypertension involves complex gene–environment interactions. Identifying high-risk subgroups for targeted intervention remains a challenge in hypertension prevention. We hypothesized that integrating the *GPX3* rs3828599 polymorphism with environmental risk factors could stratify individuals to warrant precision antioxidant-based prevention.

**Methods:**

In a rural Han Chinese case‒control investigation involving 400 hypertensive patients and 400 normotensive controls, we conducted genotyping of the *GPX3* promoter variant rs3828599, measured the serum levels of GPx-3, and evaluated the interactions between GPx-3 and metabolic and lifestyle factors. Multivariable logistic regression was employed to assess the risk of hypertension under genetic models, while gene‒environment interactions were analysed using MDR.

**Results:**

The rs3828599 C allele was found to significantly increase the risk of hypertension. In the codominant model, when the CC genotype was compared with the TT genotype, the odds ratio (OR) was 2.12 (95% CI: 1.38–3.24; *P* = 0.001), indicating an allele-dose effect, with the risk increasing in the order TT < TC < CC. The serum level of GPx-3 was significantly lower in hypertensive patients (*P* < 0.001) and displayed a genotype-dependent gradient in the order TT > TC > CC (*P* < 0.001). Serum GPx-3 levels were inversely correlated with systolic blood pressure (β=−0.424; *P* < 0.001) and diastolic blood pressure (β=−0.179; *P* < 0.001) but positively correlated with HDL-C levels (β = 0.129; *P* < 0.001). MDR analysis revealed a crucial three-way interaction: individuals who carried the rs3828599 TC/CC genotype and consumed alcohol and were of advanced age (men > 50 years or women > 65 years) had a 4.7-fold increased risk of hypertension (OR = 4.7, 95% CI: 1.827–12.092; *P* < 0.001).

**Conclusions:**

The combination of the *GPX3* rs3828599 TC/CC genotype, alcohol use, and advanced age defines a high-risk hypertension subgroup with increased oxidative stress. This precision stratification model enables targeting of antioxidant interventions to mitigate excess risk in susceptible populations.

## Introduction

Essential hypertension, a multifactorial disorder that affects more than 1.3 billion individuals globally, is a leading modifiable risk factor for cardiovascular morbidity and mortality. Uncontrolled hypertension drives irreversible end-organ damage that can lead to stroke, myocardial infarction, chronic kidney disease, and vascular dementia [1]. Its insidious onset and synergistic interactions with metabolic disorders such as obesity, dyslipidaemia, and insulin resistance amplify its societal burden, necessitating urgent advancements in prevention and precision medicine. Despite widespread pharmacological interventions, nearly half of hypertensive patients fail to achieve adequate blood pressure control, emphasizing the critical gaps in our understanding of its aetiological complexity [2]. In light of these considerations, identifying effective biomarkers that can be used in risk stratification may be advantageous for the comprehensive management of cardiovascular diseases [3].

The pathogenesis of hypertension arises from an intricate interplay between polygenic susceptibility and environmental triggers such as oxidative stress, sodium overload, and chronic inflammation. Genome-wide association studies (GWASs) have identified more than 1500 loci associated with blood pressure regulation, yet these variants collectively explain less than 20% of the heritability of hypertension, underscoring the importance of gene‒environment crosstalk [4]. Plasma glutathione peroxidase-3 (GPx-3) is encoded by the *GPX3* gene on chromosome 5q33. Unlike intracellular GPx isoforms, GPx-3 uniquely circulates in plasma. GPx-3 is also associated with various nonneoplastic diseases such as cardiovascular diseases and metabolic syndrome. Both its level of expression and its enzymatic activity undergo alterations during the pathological processes of these conditions. Nevertheless, the precise mechanisms that underlie the role of GPx-3 in cardiovascular disease warrant further investigation [5]. As a key extracellular antioxidant enzyme, it has garnered attention for its role in mitigating oxidative damage and preserving vascular endothelial function, the deterioration of which is the central mechanism in hypertension pathogenesis [6, 7]. However, interactions between *GPX3* polymorphisms, environmental factors, and metabolic indices are poorly characterized, particularly in high-risk rural populations.

This study investigated the associations among the *GPX3* rs3828599 polymorphism, serum GPx-3 levels, and hypertension risk in a high-morbidity rural Chinese cohort and explored relevant interactions between lifestyle and metabolic factors. By elucidating the gene–environment crosstalk governing GPx-3 pathways, we aim to advance personalized risk prediction and identify high-risk subgroups of individuals who are likely to benefit from targeted antioxidant intervention.

## Materials and methods

### Study population

A high hypertension morbidity rate is present in the rural areas of Fuxin County. This county is located in the northwestern part of Liaoning Province in northeastern China. A total of 800 unrelated individuals of self-reported Han Chinese ethnicity were recruited; they included 400 hypertensive patients and 400 normotensive controls. The criteria for inclusion in the hypertensive group were as follows: age > 50 years; meeting the diagnostic criteria for hypertension (defined as a resting (sitting) systolic blood pressure (SBP) of ≥ 140 mmHg and a resting (sitting) diastolic blood pressure (DBP) of ≥ 90 mmHg) or ongoing antihypertensive treatment; no clinical or biochemical manifestations indicative of secondary hypertension. Four hundred unrelated control subjects who met the following criteria were also enrolled: age > 50 years and a resting (sitting) SBP of < 140 mmHg and a DBP of < 90 mmHg. All the subjects self-identified as having four Han Chinese grandparents. Pregnant women and individuals with a history of cancer or congestive heart failure were excluded. The procedures were conducted in a manner consistent with the ethical standards set by the Human Experimentation Committee of China Medical University, and all the experimental protocols were approved by that committee. All the subjects provided written informed consent.

### Data collection and clinical measurements

Demographic data, smoking status, alcohol consumption, and medical history were collected through standardized questionnaires. Physical examination included measurement of height, weight, waist circumference (WC), and blood pressure. Body mass index (BMI) was calculated using weight in kilograms divided by height in square metres (kg/m²). Seated blood pressure was measured twice after 5 min of rest, and the average of the two measurements was recorded. Fasting venous blood samples were collected after a 12-hour fast. Serum was separated by centrifugation (3000 rpm, 20 min, 4 °C) and stored at -80 °C. Enzymatic methods conducted on an Olympus AU640 autoanalyzer (Olympus, Japan) were used to measure serum total cholesterol (TC), low-density lipoprotein cholesterol (LDL-C), high-density lipoprotein cholesterol (HDL-C), triglyceride (TG), and fasting plasma glucose (FPG) levels.

### Genotyping

**Fig. 1:**
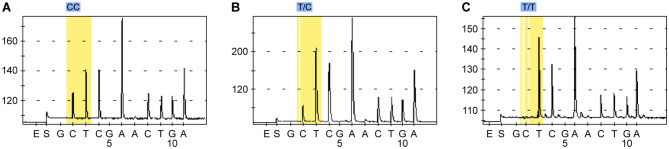
**A**. Sequencing peak map for the CC genotype. **B**. Sequencing peak map for the TC genotype. **C**. Sequencing peak map for the TT genotype

Genomic DNA was extracted from peripheral white blood cells using a TIANamp Blood DNA kit (Tiangen, Shanghai, China). Genotyping of the *GPX3* SNP rs3828599 was performed by PSQ (Qiagen, Shanghai, China). We used the PSQ assay design software to design polymerase chain reaction (PCR) amplification primers and PSQ primers. The forward primer was 5’GTTATTCATGGAGTCCACGTTCT3’, the reverse primer was Bio5’GGAGTCAGTCCCAACCTTCAG3’, and the sequencing primer was 5’CAATTGTATCTTCTTTGACC3’. The biotinylated primers were identified through the Bio preceding the oligonucleotide sequence. PCR was performed for 3 min at 95 °C followed by 45 cycles of 95 °C for 10 s, 60 °C for 20 s, and 72 °C for 30 s, with a final extension at 72 °C for 5 min. Genotyping was performed using an automated PSQ 96 instrument according to the manufacturer^’^s instructions. Genotype calls (TT, TC, and CC) were determined from the peaks visible in the pyrogram (Fig. 1). The genotyping success rate was > 98%.

### Serum GPx-3 measurement

Serum levels of GPx-3 were quantified using an enzyme-linked immunosorbent assay (ELISA) kit (Westang, Shanghai, China) in strict accordance with the manufacturer’s protocol. The intra-assay and interassay coefficients of variation were both less than 10%.

### Statistical analysis

Continuous variables are expressed as the mean ± standard deviation (SD) and were compared using Student’s t test or one-way ANOVA with post hoc testing (Tukey) where appropriate. Categorical variables are presented as counts (percentages) and were compared using the chi-square (χ²) test. Hardy‒Weinberg equilibrium (HWE) was assessed in controls using the χ² test. The association between the rs3828599 genotype and hypertension risk was evaluated using multivariable logistic regression under three genetic models: codominant (TT vs. TC vs. CC); dominant (TT vs. (TC + CC)); and recessive ((TT + TC) vs. CC). The results are presented as odds ratios (ORs) with 95% confidence intervals (CIs) adjusted for age, sex, smoking status, alcohol consumption, BMI, WC, FPG, TC, LDL-C, HDL-C and TG.

Factors associated with serum GPx-3 levels were identified using Spearman’s correlation and multiple stepwise linear regression (entry *P* ≤ 0.05, removal *P* ≥ 0.10; dependent variable: GPx-3 level; independent variables: age, sex, SBP, DBP, BMI, WC, FPG, TC, LDL-C, HDL-C, and TG).

Gene‒environment interactions were analysed using Multifactor Dimensionality Reduction (MDR) software (version 3.0.2). Environmental risk factor thresholds were selected according to the cardiovascular risk factors presented in the 2018 Chinese guidelines for the management of hypertension [8]. The variables included the rs3828599 genotype (TT = 0, TC = 1, CC = 2), sex (female = 0, male = 1), smoking status (no = 0, yes = 1), alcohol consumption (no = 0, yes = 1), FPG level (≤ 6.0 mmol/L = 0, > 6.0 mmol/L = 1), age (≤ 50 M/≤65 F = 0, > 50 M/>65 F = 1), BMI (< 28 kg/m²=0, ≥ 28 kg/m²=1), WC (< 85 cm M/<80 cm F = 0, ≥ 85 cm M/≥80 cm F = 1), LDL-C level (≤ 3.3 mmol/L = 0, > 3.3 mmol/L = 1), TG level (≤ 2.25 mmol/L = 0, > 2.25 mmol/L = 1), TC level (≤ 5.72 mmol/L = 0, > 5.72 mmol/L = 1), and HDL-C level (≥ 1.0 mmol/L = 0, < 1.0 mmol/L = 1). The best interaction model was selected on the basis of maximum testing accuracy and cross-validation consistency (CVC). Statistical significance was evaluated using 1000-fold permutation testing.

The statistical analyses were performed using SPSS 22.0 (IBM Corp., USA). Two-tailed P values < 0.05 were considered to indicate statistical significance.

## Results

### Characteristics of the participants

The characteristics of the 400 hypertensive patients and the 400 normotensive controls are summarized in Table 1. Compared with controls, hypertensive patients had significantly higher BMIs, WCs, ages, TC levels, and LDL-C levels (all *P* < 0.05). Greater proportions of hypertensive patients were male, smokers, and alcohol consumers (all *P* < 0.05). No significant differences in FPG, HDL-C, or TG levels were detected between the groups.

**Table 1:** Demographic characteristics of the study population

	Hypertension (*n* = 400)	Control (*n* = 400)	*P* value
Age (years)	65 ± 11	61 ± 9	<0.001
Sex (male/female)	164/236	128/272	0.008
SBP (mmHg)	167 ± 25	121 ± 12	<0.001
DBP (mmHg)	98 ± 12	76 ± 7	<0.001
Smoking status (yes/no)	210/190	180/220	0.034
Alcohol consumption status (yes/no)	183/217	124/276	<0.001
FPG (mmol/l)	5.89 ± 2.49	5.85 ± 1.69	0.756
WC (cm)	86.8 ± 11.0	81.3 ± 9.3	<0.001
BMI (kg/m^2^)	28.52 ± 3.66	25.53 ± 3.05	<0.001
TC (mmol/l)	5.55 ± 1.11	5.23 ± 1.09	<0.001
LDL-C (mmol/l)	3.21 ± 0.84	2.85 ± 0.77	<0.001
HDL-C (mmol/l)	1.48 ± 0.39	1.46 ± 0.37	0.107
TG (mmol/l)	1.74 ± 1.22	1.70 ± 1.13	0.623

Compared with controls, hypertensive patients had significantly higher BMI, WC, age, TC, and LDL-C and higher proportions of men, smokers, and alcohol consumers

### Genotype distribution and allele frequencies

The genotype distributions for rs3828599 in both the controls and the hypertensive patients conformed to the Hardy‒Weinberg equilibrium (HWE) (controls: PHWE = 0.067; hypertensive cases: PHWE = 0.457). The genotype and allele frequencies differed significantly between the two groups (Table 2). The frequency of the minor C allele was significantly greater in hypertensive patients (62.5%) than in controls (54.5%) (*P* = 0.001). Conversely, the frequency of the T allele was significantly lower in patients (37.5%) than in controls (45.5%). The CC genotype was more prevalent in patients than in controls (40.0% vs. 32.0% in controls; *P* = 0.006), whereas the TT genotype was less prevalent (15.0% vs. 23.0%).

**Table 2:** Genotypes and allele frequencies of *GPX3* rs3828599

	TT (%)	TC (%)	CC (%)	T (%)	C (%)	*P*-HWE^a^
Hypertension	60 (15.0)	180 (45.0)	160 (40.0)	300 (37.5)	500 (62.5)	0.457
Control	92 (23.0)	180 (45.0)	128 (32.0)	364 (45.5)	436 (54.5)	0.067
*P* value^b^	0.006			0.001		

The hypertension and control groups showed significant differences in genotype and allele frequencies. The frequency distributions in both groups were subjected to the Hardy‒Weinberg equilibrium test

^a^ Hardy‒Weinberg equilibrium test; ^b^ chi-square test

### Association of rs3828599 with hypertension risk

Multivariate logistic regression with adjustment for age, sex, smoking status, alcohol consumption status, BMI, WC, FPG, TC, LDL-C, HDL-C and TG revealed significant associations between rs3828599 and hypertension under all genetic models (Table 3). In the codominant model, both the TC genotype (OR = 1.62, 95% CI: 1.07–2.45, *P* = 0.023) and the CC genotype (OR = 2.12, 95% CI: 1.38–3.24, *P* = 0.001) were associated with significantly increased hypertension risk compared with the TT genotype. In the dominant model (TC + CC vs. TT), carriers of at least one C allele (TC or CC) had a 1.91-fold increased risk of hypertension compared with TT homozygotes (OR = 1.91, 95% CI: 1.30–2.81, *P* = 0.001). In the recessive model (CC vs. TT + TC), individuals homozygous for the C allele (CC) had a 1.74-fold increased risk of hypertension compared with carriers of at least one T allele (TT + TC) (OR = 1.74, 95% CI: 1.26–2.40, *P* = 0.001). According to the additive model, the risk of hypertension associated with the C allele was significantly greater than that associated with the T allele (OR = 1.47, 95% CI: 1.19–1.82; *P* < 0.001).

**Table 3:** Association of *GPX3* rs3828599 polymorphism with hypertension risk according to various genetic models

	Hypertension (*n*)	Control (*n*)	Unadjusted			Adjusted^a^		
			OR	95% CI	*P* value	OR	95% CI	*P* value
Codominant								
TT	60	92	1 [Reference]			1 [Reference]		
TC	180	180	1.72	1.14–2.58	0.01	1.62	1.07–2.45	0.023
CC	160	128	2.16	1.41–3.30	< 0.001	2.12	1.38–3.24	0.001
Dominant								
TT	60	92	1 [Reference]			1 [Reference]		
CC + TC	340	308	1.95	1.33–2.85	0.001	1.91	1.30–2.81	0.001
Recessive								
TC + TT	240	272	1 [Reference]			1 [Reference]		
CC	160	128	1.64	1.20–2.26	0.002	1.74	1.26–2.40	0.001
Additive								
T allele	300	364	1 [Reference]			1 [Reference]		
C allele	500	436	1.39	1.14–1.70	0.001	1.47	1.19–1.82	<0.001

Codominant model: The hypertension risk in individuals with the TT, TC, and CC genotypes increases significantly stepwise. Dominant model: The hypertension risk in individuals with the CC or TC genotype is greater than that in individuals with the TT genotype. Recessive model: The hypertension risk in individuals with the CC genotype is greater than that in individuals with the TC or TT genotypes. Additive model: The hypertension risk associated with the C allele is much greater than that associated with the T allele

^a^ Adjusted for age, sex, smoking status, alcohol consumption status, BMI, WC, FPG, TC, LDL-C, HDL-C and TG

### Association of serum GPx-3 levels with blood pressure status and rs3828599

**Fig. 2:**
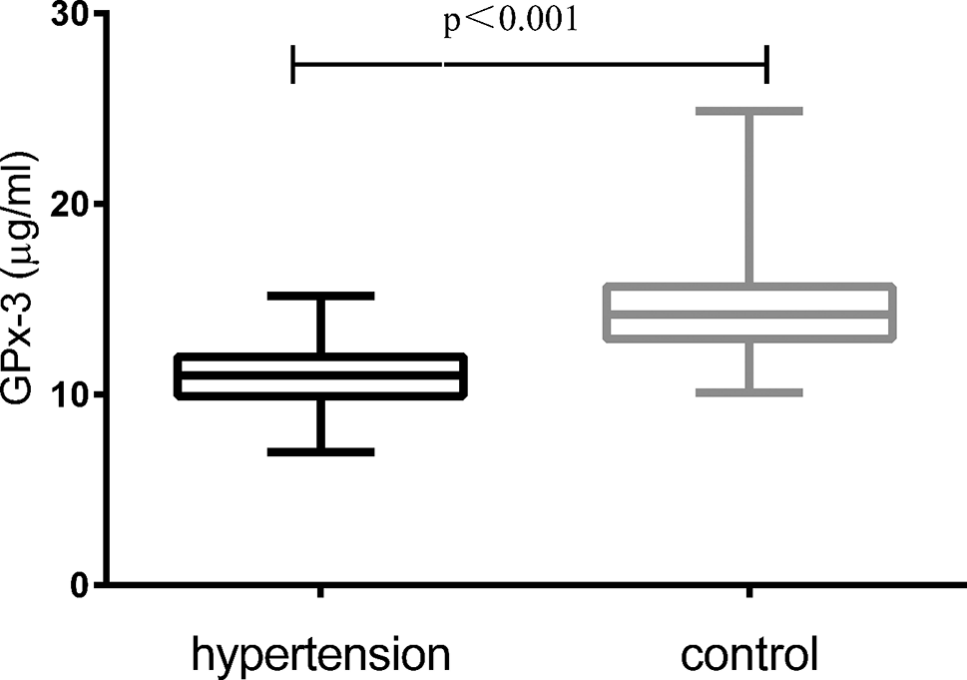
Serum GPx-3 levels were significantly lower in the hypertensive group than in the control group

**Fig. 3:**
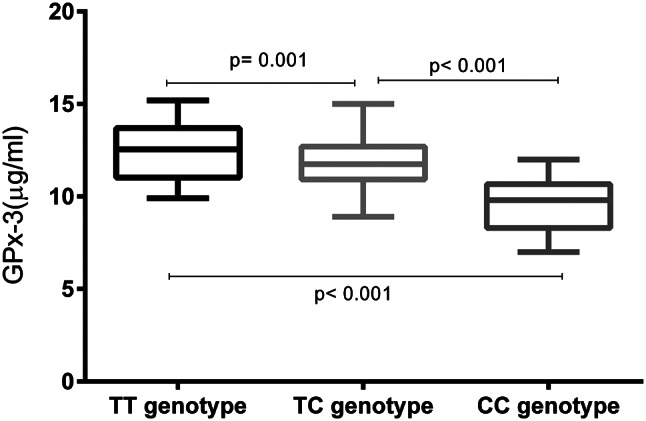
Among hypertensive individuals, serum GPx-3 levels were significantly lower in individuals with the TC or CC genotype than in those with the TT genotype

**Fig. 4:**
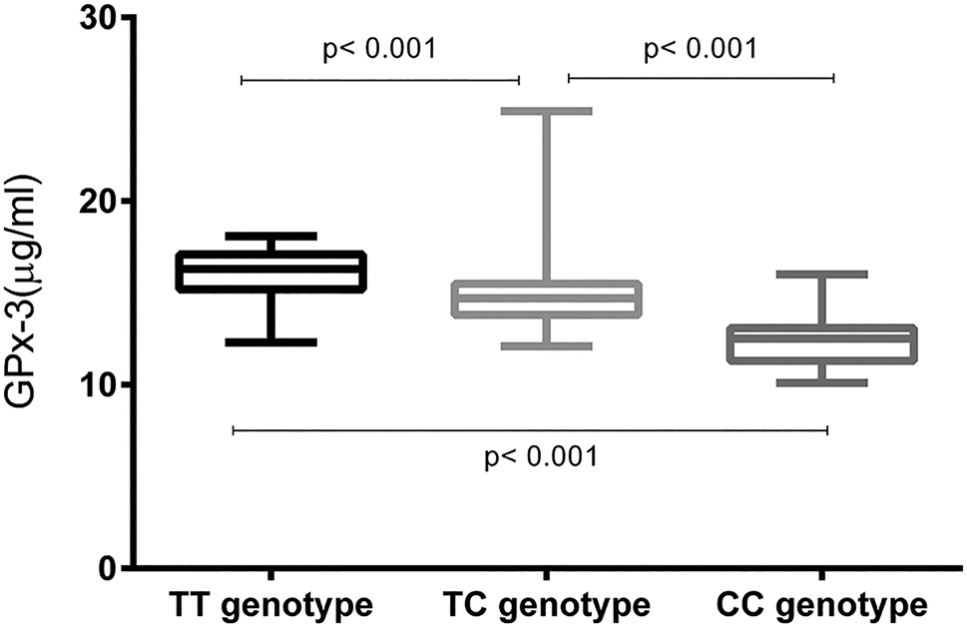
Among the controls, serum GPx-3 levels were significantly lower in individuals with the TC or CC genotype than in those with the TT genotype

Serum GPx-3 levels were markedly lower in hypertensive patients than in normotensive controls (Fig. 2; *P* < 0.001). In both the hypertensive group and the control group, serum GPx-3 levels increased significantly in a genotype-dependent manner (Figs. 3 and 4). Specifically, individuals with the TT genotype had the highest serum GPx-3 levels, those with the TC genotype had intermediate levels, and those with the CC genotype had the lowest levels (all pairwise comparisons, *P* < 0.001). This graded decrease (TT > TC > CC) was consistent both in the cases and in the controls.

### Factors associated with GPx-3 serum level

Spearman correlation analysis revealed no significant correlations between serum GPx-3 levels and sex, smoking status, or alcohol consumption (*P* > 0.05). Multiple stepwise linear regression analysis revealed that SBP (β = -0.424 *P* < 0.001), HDL-C (β = 0.129, *P* < 0.001), and DBP (β = -0.179, *P* < 0.001) were significant independent predictors of serum GPx-3 levels (R² = 0.35, *P* < 0.001; Table 4). Higher SBP and DBP were associated with lower GPx-3, while higher HDL-C was associated with higher GPx-3.

**Table 4:** Regression analysis of factors associated with serum GPx-3 levels

	B	SE	β	*P* value	95% CI for B
Constant	19.059	0.511	-	< 0.001	18.057, 20.062
SBP	-0.035	0.004	-0.424	< 0.001	-0.043, -0.027
HDL-C	0.834	0.185	0.129	< 0.001	0.471, 1.196
DBP	-0.031	0.008	-0.179	< 0.001	-0.047, -0.014

The independent variables in the analysis were age, sex, BMI, WC, SBP, DBP, FPG, TC, LDL-C, HDL-C, and TG

SBP, DBP, and HDL-C were significantly associated with serum GPx-3 levels (R² = 0.35, *P* < 0.001)

### Analysis of Gene‒Environment interactions

MDR analysis revealed a significant 3-factor interaction model comprising the rs3828599 genotype (TC/CC), alcohol consumption (yes), and advanced age ( men > 50 years or women > 65 years) (Table 5). This model had the maximum testing accuracy (68.37%) and maximum cross-validation consistency (10/10). Permutation testing confirmed the significance of this interaction (*P* < 0.001). Compared with individuals without this combination, individuals with the combined high-risk profile (rs3828599 TC/CC + alcohol consumption + advanced age) had a 4.7-fold increased risk of hypertension (OR = 4.7, 95% CI: 1.827–12.092).

**Table 5:** MDR analysis of Gene‒Environment interactions for hypertension risk

Factor No. and Combination	Testing accuracy (%)	CV consistency		*P* value	OR (95% CI)
1-factor: age	63.50	10/10	0.016		3.036 (1.220–7.557)
2-factor: age, BMI	64.38	8/10	0.010		3.272 (1.309–8.178)
3-factor: rs3828599, age, alcohol consumption	68.37	10/10	0.001		4.7 (1.827–12.092)

The combination of the factors rs3828599 TC/CC, alcohol consumption and advanced age was associated with a significantly higher risk of hypertension

## Discussion

### Demographic risk factors and susceptibility to hypertension

Numerous studies have shown that high-quality and easily performed biomarker detection can be beneficial for the early diagnosis, prevention, and monitoring of diseases [9–12]. Our study revealed significant associations between elevated anthropometric and metabolic indices, including BMI, WC, TC, and LDL-C, and hypertension risk in a rural Han Chinese cohort. Compared with controls, hypertensive patients had higher mean BMIs (28.52 kg/m² vs. 25.53 kg/m²) and WCs (86.8 cm vs. 81.3 cm)consistent with evidence linking central obesity and dyslipidaemia to endothelial dysfunction and oxidative stress [1]. Male sex, smoking, and alcohol consumption were also more prevalent among hypertensive individuals, consistent with known sex-specific and lifestyle-driven hypertension mechanisms. Smoking-induced generation of reactive oxygen species (ROS) and alcohol-related vasoconstriction may synergize with genetic susceptibility to exacerbate blood pressure elevation [13]. These findings reinforce the role of modifiable metabolic and behavioural factors in the pathogenesis of hypertension, particularly in populations with high rates of obesity and traditional risk behaviours.

### Individual variability in prevention efficacy and genetic susceptibility

Although lifestyle modifications have certain benefits, our data emphasize their inherent limitations. Specifically, not all individuals can achieve the same level of risk reduction through lifestyle modification.This reflects the interaction between genetic predisposition and environmental exposure. In the era of precision medicine, the interaction between genes and the environment should be fully considered in the prevention and treatment of hypertension [14]. Emerging evidence emphasizes that individualized risk stratification that integrates genetic predisposition, epigenetic modifications, and measurement of biomarkers of oxidative stress could shift preventive strategies to include individuals who are in the prehypertensive stage, particularly in populations with inherited vulnerabilities [15]. Genetic identification of individual oxidative stress signatures, such as GPx gene polymorphisms, and the evaluation of biomarkers could facilitate the development of precision medicine strategies that are tailored to individual oxidative stress profiles [16]. Our study revealed that heterogeneity in hypertension risk is exemplified by the *GPX3* rs3828599 polymorphism. Our key finding is the strong association between the presence of the rs3828599 C allele and increased susceptibility to hypertension. Compared with TT homozygotes, individuals carrying the CC genotype had more than twice the risk of hypertension, even after adjustment for major covariates such as age, sex, BMI, serum lipid levels, and lifestyle factors. The codominant model showed a clear allele-dose effect (TT < TC < CC risk), suggesting a potential functional impact of this SNP on GPx-3 biology. Previous research has shown that in the general Thai population, the GPX3 rs3828599 polymorphism is independently associated with the incidence of hypertension, elevated triglyceride (TG) levels, and low high-density lipoprotein cholesterol (HDL-C) levels. Moreover, when other antioxidant-related gene polymorphisms (SOD2, SOD3, PON1, and GSTT1) are present, GPX3 rs3828599 is more strongly associated with the incidence of hypertension and is positively associated with coronary artery disease and with the severity of coronary atherosclerosis [17, 18]. An association analysis of 161 SNPS of 10 candidate genes, including *GPX3*, in a Japanese population with hypertension revealed that no polymorphisms correlated significantly with blood pressure level [19]. The population-specific disparities observed in these associations accentuate the influence of genetic ancestry and behavioural context. In rural Chinese cohorts, in which selenium deficiency and high-sodium diets are prevalent, the interactions between *GPX3* variants and lifestyle factors may disproportionately increase oxidative stress. This, in turn, exacerbates genetic susceptibility to hypertension. Furthermore, genetic susceptibility plays a role in modulating the effectiveness of interventions. For example, individuals with abnormal GPx expression may continue to display elevated oxidative stress levels even after lifestyle modifications. This is due to inherent compromise of their antioxidant defences [20]. Thus, although lifestyle interventions remain foundational, genetic profiling could help identify subgroups requiring adjunct therapies such as antioxidant supplementation or targeted pharmacotherapy.

### rs3828599 Genotype, GPx-3 protein Levels, and hypertensive mechanisms

We found that serum GPx-3 levels were significantly decreased in hypertensive patients compared with controls. Conversely, the T allele of GPX3 rs3828599 increased the circulating protein expression level of GPx-3, whereas the C allele of rs3828599 decreased it. The genotype-dependent gradient in serum GPx-3 level (TT > TC > CC) that was observed in both cases and controls highlights the functional influence of rs3828599 on enzyme activity. This case‒control study provides robust evidence that the rs3828599 C allele is associated both with reduced circulating GPx-3 levels and with an increased risk of hypertension in a Chinese Han population. Although the exact functional implications of rs3828599 have yet to be comprehensively elucidated, its position within the promoter region of the gene implies that it may affect transcription factor binding, mRNA stability, or splicing. This could ultimately influence the expression or secretion of the enzyme [21]. The promoter region of the human *GPX3* gene harbours an antioxidant response element (ARE) and a metal response element (MRE). These elements are sensitive to regulation by ROS and antioxidants and can significantly increase transcriptional activity [22]. A reduction in GPx-3 levels compromises the glutathione redox cycle. This disruption leads to an imbalance between the production and the elimination of ROS. This imbalance triggers the activation of proinflammatory pathways, including the NF-κB pathway; consequently, angiotensin II signalling is upregulated, and vasoconstriction and sodium retention are further exacerbated [23]. Our observation that serum GPx-3 levels correlate inversely with systolic and diastolic blood pressure strengthens the evidence for a role of this enzyme as both a biomarker and a mediator of hypertensive pathology.

The significant positive correlation between HDL-C and GPx-3 levels was intriguing. As an indicator of lipid oxidation, a cross-sectional study showed that a decrease in the LDL/HDL index was associated with decreased GPx-3 level and a decrease in the oxLDL/HDL index [24]. According to detailed endothelial cell kinetic model analysis, glutathione (GSH) and GPx levels are depleted or maintained in response to oxidative stress conditions in individuals with vascular pathologies [25], and a nested case‒control prospective study showed that serum GPx-3 activity correlates inversely with mortality due to coronary vascular disease in subjects with low HDL-C levels and is independent of conventional risk factors [26]. The potential synergistic relationship between HDL and GPx-3 in mitigating vascular oxidative stress warrants further investigation.

### Gene‒environment interactions and personalized prevention strategies

Gene‒environment interactions (G×E) involve not only simple additions but also dynamic amplification or buffering effects. To identify effective treatment strategies for complex diseases, precision medicine necessitates comprehensive evaluation of the patient’s genomic profile and environmental exposure history [27]. MDR analysis revealed significant three-way interactions among rs3828599 risk genotype (TC/CC), alcohol consumption, and advanced age (>50 years for men and >65 years for women). This interaction increased the risk of hypertension by 4.7-fold. Specifically, the genetic risk conferred by the C allele was potentiated by alcohol consumption and advanced age. This synergy indicates that environmental stressors such as alcohol-induced oxidative damage act in combination with genetic susceptibility to overpower endogenous antioxidant defences. Our study population was drawn from a region in which zinc deficiency is common and was characterized by the prevalence of a high-salt diet, significant alcohol consumption, and a substantial incidence of hypertension [28, 29]. Ageing is among the prominent risk factors for hypertension. As ageing progresses, the body’s capacity to efficiently produce and utilize antioxidants gradually declines. Consequently, damage due to oxidative stress occurs. Ageing is also intricately associated with inflammation. Persistent low-grade inflammation can further intensify the development of hypertension and other age-related disorders. Overall, the intricate interplay among ageing, reduced antioxidant efficiency, increased oxidative stress damage, and inflammation poses a formidable challenge to maintaining cardiovascular health and contributes substantially to an increased risk of hypertension [30, 31]. Cross-sectional and prospective investigations have tracked the development of hypertension in relation to changes in alcohol consumption over time. One study revealed that the interaction between alcohol consumption and the CYP11B2 risk genotype is associated with a threefold increase in the risk of hypertension [27]. Furthermore, alcohol consumption can lead to malnutrition and a concomitant deficiency of trace elements, including selenium [32]. After pigs were fed diets containing various amounts of selenium for 16 weeks, the level of expression of GPx-3 mRNA in the spleens of the Se-deficient animals decreased significantly compared with that of the animals in the other groups, suggesting that dietary Se levels can affect transcription of the GPx-3 gene in the spleen [33]. The mRNA levels of GPx-3 are significantly downregulated in mice receiving total parenteral nutrition [34]. These findings indicate that dietary habits, nutritional choices, and nutritional status can influence the expression of GPx-3 in the context of zinc deficiency.

The findings discussed above underscore the imperative need for precision prevention frameworks that integrate genetic and environmental risk profiling. For instance, *GPX3* rs3828599 C allele carriers with concurrent alcohol use could be prioritized for intensive lifestyle coaching and selenium supplementation to increase GPx-3 activity. Furthermore, integrating *GPX3* genotyping into risk scores based on traditional risk factors may improve predictive accuracy and facilitate the early identification of high-risk subgroups. However, population-specific variations in genetic architecture and environmental exposure necessitate regionally tailored approaches. China’s vast territory results in diverse living habits across regions, and this in turn contributes to variations in hypertension risk factors. Therefore, the implementation of individualized intervention strategies that consider both genetic and traditional risk factors and are customized to fit specific regional contexts is likely to result in greater benefits [35]. In rural areas of China, where selenium deficiency, high-sodium diets, and excessive alcohol intake are prevalent, interventions centred on the three-way interaction model may yield disproportionately substantial benefits.

### Study strengths and limitations

This study has several notable strengths. First, it features a well-phenotyped case‒control design, which provides a solid foundation for the investigation. Appropriate adjustments were made for the major confounders, enhancing the validity of the results. The direct measurement of serum GPx-3 levels not only revealed a genotype‒phenotype correlation but also contributes to understanding of the biological mechanisms involved. The identification of significant gene‒environment interactions using MDR provides a basis for the comprehensive and precise prevention of hypertension.

However, this study has certain limitations. First, the case‒control design inherently lacks the ability to establish causality, and this restricts the inferences that can be drawn regarding cause‒and‒effect relationships. In future study, it will be crucial to carry out antioxidant intervention clinical trials in high-risk subgroups to validate the results. Second, the research focused on a single SNP. Comprehensive haplotype or sequencing analyses might offer more profound insights into the genetic architecture related to the studied phenomenon. Third, the study population was ethnically homogeneous and consisted of Han Chinese individuals. As such, the generalizability of the findings to other populations requires validation.

## Conclusion

This research comprehensively elucidates the intricate synergistic interplay among *GPX3* rs3828599-related genetic susceptibility, redox imbalance, and environmental risk factors in the pathogenesis of hypertension. The *GPX3* rs3828599 C allele significantly elevates the risk of hypertension by reducing the activity of GPx-3 and is involved in crucial gene–environment interactions, especially in relation to alcohol consumption and advanced age, resulting in a 4.7-fold increase in the risk. The key findings are as follows. First, a high-risk subgroup was identified: individuals who carry the *GPX3* rs3828599 C allele, who consume alcohol, and who are over 50 years of age (in the case of men) or 65 years of age (in the case of women) may benefit from targeted antioxidant interventions. Second, emphasis should be placed on extracellular antioxidant defence, considering that GPx-3 functions both as a biomarker and as a mediator. Third, future studies should focus on the functional validation of rs3828599 and should explore other GPX3 variants and implement antioxidant trials in genetically susceptible cohorts.

## Data Availability

The data collected in this study are available from the corresponding author upon reasonable request.
